# Relationship Between Serum Concentration of Adrenomedullin and Myocardial Ischemic T Wave Changes in Patients With Lung Cancer

**DOI:** 10.3389/fcvm.2022.836993

**Published:** 2022-03-07

**Authors:** Chen Wu, Da-wei Lin, Yi-wen Jiang, Feng Jiang, Zhao-xia Wang, Yao-sheng Wang

**Affiliations:** ^1^Department of Cardiology, Xinhua Hospital Affiliated to Shanghai Jiao Tong University School of Medicine, Shanghai, China; ^2^Clinical Research and Innovation Unit, Xinhua Hospital Affiliated to Shanghai Jiao Tong University School of Medicine, Shanghai, China; ^3^Department of Cardiology, Xinhua Hospital Affiliated to Shanghai Jiao Tong University School of Medicine Chongming Branch, Shanghai, China

**Keywords:** adrenomedullin (ADM), T wave inversion or flat, myocardial ischemia, cardiovascular diseases (CVDs), lung cancer

## Abstract

**Background:**

Patients with lung cancer are at increased risk for the development of cardiovascular diseases. Molecular markers for early diagnosis of cardiac ischemia are of great significance for the early prevention of cardiovascular events in patients with lung cancer. By evaluating the relationship between adrenomedullin (ADM) and myocardial ischemic T wave changes, the clinical value of circulating ADM as a predictor of myocardial ischemia in patients with lung cancer is confirmed.

**Methods:**

We enrolled patients with lung cancer and healthy people from 2019 to 2021 and extracted a detailed ECG parameter. After adjustment for potential confounders, logistic regression was used to assess the association of clinical data. We performed analyses on differences in T wave between patients with lung cancer and healthy people, and the relationship between T wave and ADM among patients with lung cancer. Receiver operator characteristic (ROC) curves were drawn to confirm the diagnostic value of biomarkers.

**Results:**

After adjusting for potential confounders, the incidence of T wave inversion or flattening in patients with lung cancer was higher than in healthy people (OR: 3.3228, *P* = 0.02). Also, further analysis of the data of lung cancer patients revealed that the ADM in lung cancer patients with T wave inversion or flat was higher than those with normal T wave (189.8 ± 51.9 vs. 131.9 ± 38.4, *p* < 0.001). The area under the ROC curve was 0.8137.

**Conclusion:**

Among the patients with lung cancer, serum ADM concentration is associated with the incidence of the abnormal T wave. ADM might be a potentially valuable predictor for heart ischemia in patients with lung cancer.

## Introduction

Cancer and cardiovascular diseases (CVDs) are two major killers among humans across the world. Cancer incidence and mortality have been rising steadily over the past several decades (1). Recent data have indicated that CVDs have become a leading cause of morbidity and mortality among cancer survivors (2). This is largely because of tumor invasiveness (like direct compression or infiltration and several inflammatory factors) and cancer treatment-related cardiotoxicity (3). Prevention of CVDs has important clinical implications for cancer survivors.

Among all cancers worldwide, lung cancer is the most commonly diagnosed with the highest mortality, and the burden of this disease is considerable. In China, it was estimated that 2.09 million new cases of lung cancer occurred globally in 2018, ranking first among all cancer types (4, 5). Anatomically, the lung is adjacent to the heart, and pulmonary circulation is a closed circuit between the right side of the heart (right ventricle, RV) and the lungs. A lung tumor releases pro-inflammatory cytokines (TNF-α, IL-1β, IL-6, and IFN-γ), chemokines, and soluble factors into the blood. These substances are then returned to the heart *via* pulmonary circulation and directly elicit catabolic responses in the heart (6, 7). However, the sensitivity and specificity of these indicators were not high enough to be specific biomarkers of CVDs. In addition to the properties of the tumor itself, clinical evidence has also shown that long term anti-cancer therapy can lead to a persistently increased CVDs risk (8). Platinum-based chemotherapy, microtubular agents, ALK inhibitors, endothelial growth factor receptor (EGFR) inhibitors, and immunotherapy represent principal clinical options for lung cancer therapy (9–11). All of these drugs have been shown to result in cardiotoxicity. Radiotherapy also plays a critical role in the management of early- or locally advanced-stage lung cancer. During radiotherapy for lung cancer, radiation often involves surrounding tissues and organs, especially the heart. Prolonged radiation exposure leads to capillary network and myocardium damage, eventually contributing to the development of myocardial, heart valve, and cusp fibrosis (12). Given that lung cancer has been known to have a higher risk of cardiovascular events, potential biomarkers to indicate susceptibility to heart ischemia need to be identified (13).

Adrenomedullin (ADM) is a 52-amino acid peptide hormone that was originally identified in extracts of human pheochromocytoma (14). The expression of ADM is widely distributed throughout the cardiovascular system and has been identified in blood vessels and the heart. There is increasing evidence that elevated circulating and tissue levels of ADM are associated with cardiovascular events. In a rat myocardial infarction (MI) model, the use of ADM has shown beneficial effects on survival and ameliorated the progression of left ventricular remodeling and heart failure (15, 16). A recent study revealed that ADM induces beneficial hemodynamic, hormonal, and myocardial changes, which can improve outcomes of patients with HF (17). In addition, various studies have indicated that imbalance in the expression of ADM is closely related to the occurrence and development of CVDs (18). ADM can be considered as biomarkers of various CVDs diagnoses, prognoses, and surveillance. The serum level of ADM has a significant positive correlation with the severity of CVDs. Because of the stability of ADM in whole blood, serum, or plasma, numerous studies have speculated that serum ADM levels can be used as an early warning indicator of cardiac ischemia.

## Materials and Methods

### Study Subjects

We performed a cross-sectional study that included patients with lung cancer and healthy populations who visited the Chongming branch of Xin Hua Hospital from 2019 to 2021 and extracted detailed information on ECG. The data were extracted from electronic medical records in the Chongming branch of Xin Hua Hospital. In this way, we identified 67 patients with lung cancer and 81 healthy people. Exclusion criteria were as follows: (1) abnormal thyroid function, (2) congenital heart disease, (3) a history of heart surgery, (4) severe valvular heart disease, (5) myocardial diseases, (6) coronary heart disease, (7) arrhythmia, and (8) diabetes. The local ethical committee approved the protocol of the study and waived the need for informed consent because of the observational characteristic of the study, and the study followed the principles outlined in the Declaration of Helsinki.

### Electrocardiogram Recordings

**Figure 1:**
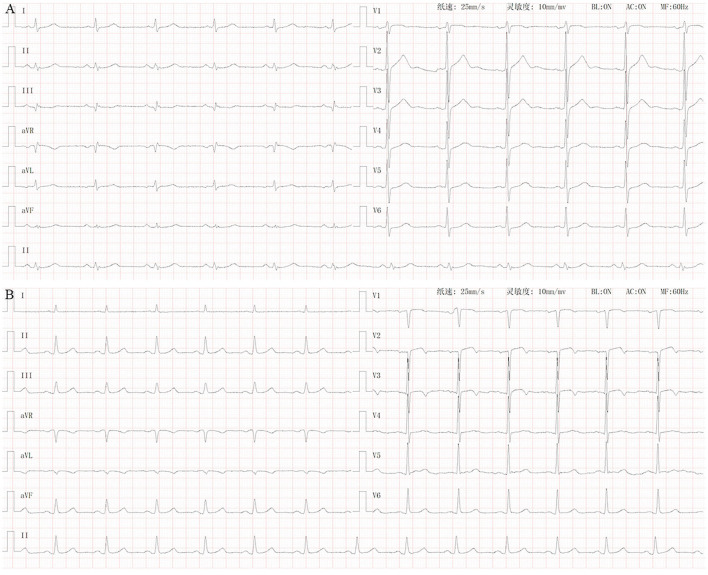
Normal ECG vs. ECG of patients with lung cancer with T wave changes. **(A)** Normal ECG. **(B)** ECG of lung cancer patients with T wave inversion or flattening (I, aVL, V3, V4, and V5).

Standard 10-s 12-lead ECGs of all the subjects were recorded. Inverted T waves with an amplitude ≥0.1 mV were considered to be abnormal, whereas those with peak amplitude between +1 and −1 mm were considered to be flat. All procedures and operations were performed by experienced cardiologists (Figure 1).

### Serum Collection

Patients with lung cancer routinely underwent routine blood tests over the course of their treatment and follow-up stages. Residual blood from clinical tests was collected during these stages. Whole blood samples were obtained and kept at room temperature for 2 h or placed at 4°C overnight. Serum samples were collected by centrifugation (20 min at 1,000 × *g*), preserved, and stored at −80°C before analysis.

### Enzyme-Linked Immunosorbent Assay

The frozen serum samples were used to measure the serum levels of ADM. ELISA was performed with commercial ELISA kits (Shanghai Tongwei Industrial Co. Ltd., TW10166) according to the manufacturer's instructions. Each independent experiment was performed three times.

### Covariates

For each study subject, we extracted data on demographic information, physical examination, history and comorbidity, concomitants, laboratory tests, and prescribed related medications from electronic medical records, and conducted a retrospective review. All the data were checked and validated; if there were any outliers, we manually checked the original dataset.

### Statistical Analysis

Continuous variables were expressed as mean values ± SD, whereas categorical variables were expressed as frequencies between subjects with and without T wave inversion or flattening. Kolmogorov-Smirnov test was performed to examine whether continuous variables were normally distributed, and analyses were performed by Student's *t*-test. Associations between categorical variables were tested by Pearson's χ2 test. Logistics regression analysis was performed to evaluate risk factors for T wave inversion or flattening, and ROC analysis was performed to assess the efficiency of distinguishing patients with lung cancer with myocardial ischemic T wave change (T wave inversion or flattening) from patients without.

All the statistical analyses were performed using the STATA 15.1 software. A *P* < 0.05 was considered statistically significant.

## Results

### Clinical Characteristics of the Subjects

The general characteristics of patients with lung cancer (*n* = 67) and health check-up people (*n* = 82), including gender, age, being older, history of drug use, and related risk factors are summarized in Table 1. Among all the enrolled individuals, 9 (11%) healthy people had T wave inversion or flattening, while 16 (23.9%) patients with lung cancer had T wave inversion or flattening. The rate of occurrence of T wave inversion or flattening in patients with lung cancer was higher than that in the healthy control population (23.9% vs. 11%, *p* = 0.036), and smokers among them were markedly more than healthy subjects (44.8% vs. 8.5%, *p* < 0.001). Baseline characteristics of the patients with lung cancer stratified by presence/absence of T wave inversion or flattening are summarized in Table 2. T wave inversion or flattening was more likely to be detected in female patients with lung cancer (47 vs. 14%, *p* = 0.01). Compared to patients with normal T wave, patients with T wave change exhibited a higher proportion of pulmonary nodules (56.3 vs. 17.6%, *p* = 0.002) and a lower proportion of smokers (19 vs. 53%, *p* = 0.016).

**Table 1:** Characteristics of checkup people and patients with lung cancer.

**Characteristics**	**Health people *n* = 82**	**Lung cancer patients *n* = 67**	***P*-value**
Male, n	63 (76.8%)	53 (79.1%)	0.74
Female	19 (23.2%)	14 (20.9%)	
Age, years	71.3 ± 7.0	69.8 ± 8.1	0.23
Older, n (%)	24 (29.2%)	15 (22.4%)	0.34
Current smoking, n	7 (8.5%)	30 (44.8%)	<0.001
Alcohol consumption, n	8 (9.8%)	13 (19.4%)	0.092
Hypertension, n	28 (34.1%)	33 (49.3%)	0.062
CAC, n	30 (36.7%)	8 (11.9%)	0.11
Pulmonary nodules, n	19 (23.2%)	18 (26.9%)	0.70
β-blocker, n	9 (11.0%)	4 (6.0%)	0.28
CCB, n	9 (11.0%)	4 (6.0%)	0.28
ACEI, n	7 (8.5%)	2 (3.0%)	0.16
ARB, n	4 (4.9%)	7 (10.4%)	0.20
T wave inversion or flat, n	9 (11.0%)	16 (23.9%)	0.036

*Older, age ≥ 75 years; CCB, calcium channel blocker; ACEI, angiotensin-converting enzyme inhibitor; ARB, angiotensin receptor blockers; CAC, coronary artery calcification. Value was given as mean ± SD, or frequency (percentage)*.

**Table 2:** Characteristics of lung cancer patients with T wave inversion or flattening.

**Characteristics**	**T wave inversion or flat *n* = 16**	**Normal T wave *n* = 51**	***P*-value**
Male, n	9 (53%)	44 (86%)	0.010
Female	7 (47%)	7 (14%)	
Age, years	69.7 ± 9.8	69.9 ± 7.6	0.93
Older, n	3 (19.0%)	12 (24.0%)	0.69
Current smoking, n	3 (19.0%)	27 (53.0%)	0.016
Alcohol consumption, n	1 (6.0%)	12 (24.0%)	0.13
Hypertension, n	9 (56.0%)	24 (47.0%)	0.52
CAC	5 (31.3%)	3 (5.9%)	0.73
Pulmonary nodules, n	9 (56.3%)	9 (17.6%)	0.002
β-blocker, n	1 (6.0%)	3 (6.0%)	0.96
CCB, n	0 (0%)	4 (7.8%)	0.25
ACEI, n	0 (0%)	2 (3.9%)	0.42
ARB, n	2 (12%)	0 (0%)	
Chemotherapy, n	8 (50.0%)	30 (59.0%)	0.53
Lung cancer operation, n	2 (12.0%)	3 (6.0%)	0.38
Pleural effusion, n	4 (25.0%)	5 (10.0%)	0.15

*Older, age ≥ 75 years; CAC, coronary artery calcification; CCB, calcium channel blocker; ACEI, angiotensin-converting enzyme inhibitor; ARB, angiotensin receptor blockers. Value was given as mean ± SD, or frequency (percentage)*.

### T Wave Inversion or Flat in Healthy People and Patients With Lung Cancer

By using Logistics regression analysis, lung cancer patients were more likely to observe T wave inversion or flat compared with healthy controls…and medication use [angiotensin-converting enzyme inhibitor (ACEI), angiotensin receptor blockers (ARB), β-blocker, and calcium channel blocker (CCB)].

### Serum ADM Levels in Patients With Lung Cancer With or Without T Wave Inversion or Flattening

**Figure 2:**
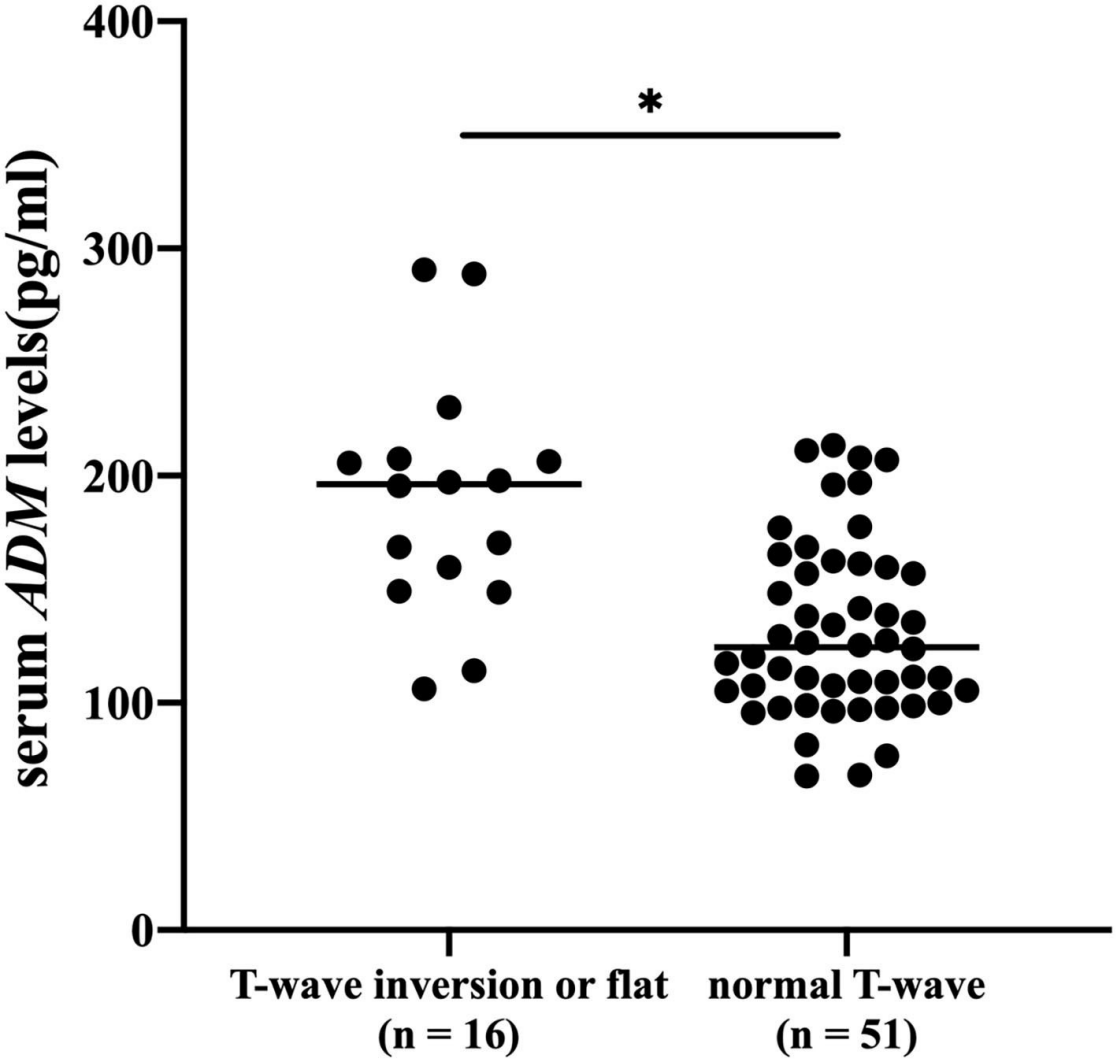
Serum levels of adrenomedullin (ADM) (pg/ml) were measured in patients with lung cancer with or without T wave inversion or flattening. *p < 0.05.

Enzyme-linked immunosorbent assay (ELISA) was performed to assess serum levels of ADM. The level of ADM in serum was significantly higher in patients with T wave inversion or flattening than in those without, as shown in Figure 2. Average serum levels of ADM were 189.8 ± 51.9 in patients with abnormal T wave and 131.9 ± 38.4 in patients without. A logistic regression model indicated that ADM was associated with the incidence of T wave inversion or flattening [odds ratio (OR): 1.4102, *p* = 0.036, 95% confidence interval (CI): 1.0232–1.9437] after being adjusted for all the accepted confounders: gender, age, being older, hypertension, lung cancer operation, pleural effusion, pulmonary nodules, current smoker, alcohol consumption, and history of drug use (chemotherapy drug, ACEIs, ARBs, β-blockers, and CCBs).

**Figure 3:**
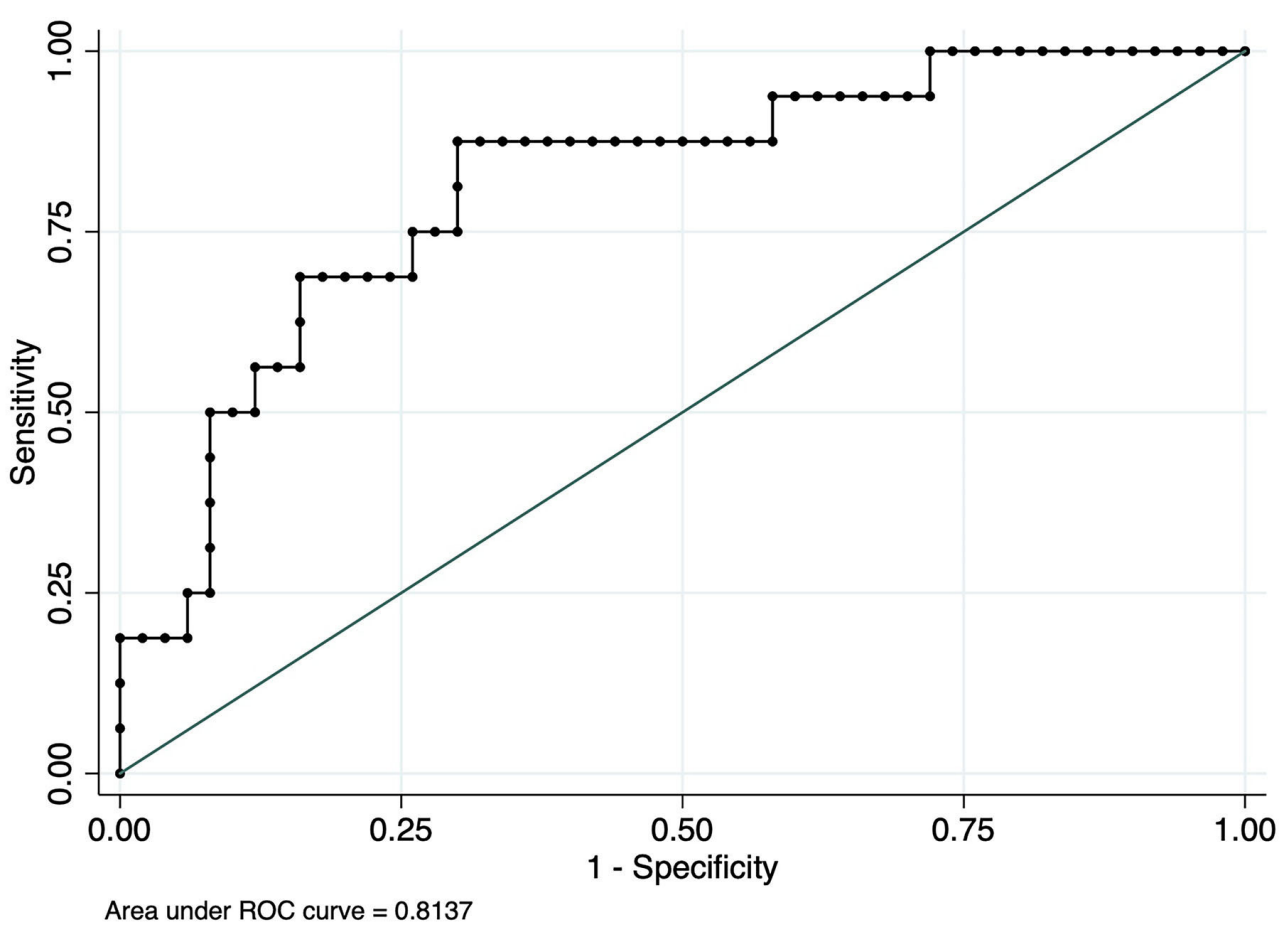
Receiver operator characteristic (ROC) curve analysis using serum ADM to distinguish myocardial ischemia-related T wave in patients with lung cancer.

To investigate the relationship between ADM and myocardial ischemic-related T wave alteration in patients with lung cancer, ROC analyses were performed to evaluate the diagnostic ability of ADM. As shown in Figure 3, a ROC curve for ADM is plotted to judge the reliability of the model and reflect the distinction between patients with or without T wave inversion or flattening, with an area under the curve (AUC) of.8137 (95% CI:0.69426–0.93324). ADM displayed acceptable sensitivity and specificity for the prediction of myocardial ischemia in patients with lung cancer.

## Discussion

We first demonstrated the reciprocal relationship between myocardial ischemia and lung cancer. We performed logistic regression analyses and found that the incidence of the ischemic T wave in patients with lung cancer was two times that in healthy subjects.

Myocardial ischemia is a chronic pathological condition, in which the heart receives oxygen slightly, and it is also an early pathological process during the occurrence and development of cancer-induced and anticancer-therapy induced CVDs (19). Prolonged ischemia leads to loss of heart contractility because of poor proliferation capability of myocardial cells, which consequently contributes to CVDs, including heart failure, arrhythmia, MI, etc. (20). Clinically, ECG is the most used method for monitoring myocardial ischemia. The leading ECG abnormality in myocardial ischemia is characterized by abnormal ventricular repolarization. Therefore, the QRS complex, which represents ventricular depolarization, is not the best index to diagnose ischemia. The T wave of the electrocardiogram is generated by transmural differences in action potential waveform. Under normal conditions, the T wave in human beings is usually positive except in aVR and often in V1. Causes of T wave inversion and flattening are related to abnormal depolarization. In previous studies, negative T wave in ischemic heart disease represents ischemia and is located in the subepicardial area (19). In contrast to the ST segment, acute ongoing ischemia does not cause a negative T wave. A negative or flat T wave appears when acute ongoing ischemia is vanishing or has already transitioned into chronic cardiac ischemia (21). Likewise, cancer-related or anticancer therapy-induced cardiac ischemia is a chronic process, which is consistent with pathological processes of the negative or flat T wave.

ECG signals collected from the surface of the human body inevitably suffer from EMG interference, power frequency interference, and some drugs. Therefore, the serodiagnostic method would be a better choice. ADM is a vasoactive peptide whose main functions are vasodilatation and maintaining vascular integrity and decreasing vascular leakage. A recent study has suggested that circulating ADM is a useful biomarker for the diagnosis of CVDs. It has been demonstrated that serum ADM levels were elevated in heart failure (HF) patients, and its change in response to the pathophysiologic changes of HF. After adjusting for other biomarkers, including natriuretic peptide (BNP) and atrial natriuretic peptide (ANP), etc., serum ADM remained a strong predictor of HF. A subsequent clinical study has shown a strong association between high levels of serum ADM and the prognosis of HF (22). Higher levels of ADM reflect residual tissue congestion and are independently associated with a higher risk of hospital readmissions (14, 23). Moreover, one study found elevated serum levels of ADM in patients with acute myocardial infarction (AMI). In the acute stage of AMI, circulating levels of ADM elevate and peak within 24 h and gradually decrease over a 3-week period (24). However, few studies have focused on ADM related to cardiac ischemia, especially among patients with cancer who face a high lifetime risk of CVDs.

In this study, we sought to find a useful biomarker for the early detection of myocardial ischemia in patients with lung cancer. By using logistics regression analysis, we adjusted for influencing factors including gender, age, history of cardiovascular and cerebrovascular diseases, lifestyle risk factors, and history of related drug therapy; the results indicated an independent positive association between serum ADM level and myocardial ischemia-related ECG change (T wave inversion or flattening) among the patients with lung cancer. Overall, our results demonstrated that patients with lung cancer with higher ADM levels have a tendency to increase the risk of myocardial ischemia compared to lung cancer patients with lower ADM levels. The current clinical guidelines do not offer specific recommendations for heart monitoring or the use of cardioprotective drugs in patients with lung cancer. Therefore, ADM may be used as a potential indicator to guide heart monitor and prevention strategies for patients with lung cancer.

## Conclusion

Elevated serum ADM levels in patients with lung cancer are correlated with increased incidence of the negative or flat T wave, which has been implicated in chronic myocardial ischemia. ADM may be an early biomarker of lung cancer-related myocardial ischemia.

## Limitations

There are few limitations to this study. First, the study, with a retrospective study design, suffered from a relatively small sample size due to single-center data and relatively strict exclusion criteria. Further large-scale studies are needed to validate the clinical utility of ADM as a practical biomarker for myocardial ischemia patients with lung cancer. In addition, this study is a cross-sectional case-control one, and prospective follow-up studies should be conducted to better assess the predictive value of biomarkers for patients with lung cancer. Third, factors leading to the occurrence of T wave inversion or flattening are very complex. Although we have taken the confounding factors as comprehensively as possible, there would still be some other unmeasured confounders causing bias in the propensity-score-match cohort. Moreover, the type of lung cancer may also affect the incidence rate of myocardial ischemia in patients with lung cancer. We did not perform data analysis for different types of lung cancer because of insufficient subgroup data within each type of lung cancer patients.

## Data Availability Statement

The original contributions presented in the study are included in the article/supplementary material, further inquiries can be directed to the corresponding author/s.

## Ethics Statement

The studies involving human participants were reviewed and approved by Xin Hua Hospital, Affiliated to Shanghai Jiao Tong University School of Medicine. The patients/participants provided their written informed consent to participate in this study.

## Author Contributions

Y-sW, Z-xW, CW, and D-wL: conception and design. FJ and Y-sW: administrative support. CW, D-wL, and Y-wJ: provision of study materials or patients. FJ, CW, D-wL, and Y-wJ: collection and assembly of data. Z-xW, CW, D-wL, and Y-wJ: data analysis and interpretation. All authors wrote the manuscript, approved the final manuscript, and have read and agreed to the published version of the manuscript.

## Funding

This work was supported by grants from the National Natural Science Foundation of China (grant no. 81974022), Shanghai Municipal Health Commission (grant no. 201940206), and the Chongming Branch of Xinhua Hospital Affiliated with the School of Medicine, Shanghai Jiao Tong University.

## Conflict of Interest

The authors declare that the research was conducted in the absence of any commercial or financial relationships that could be construed as a potential conflict of interest.

## Publisher's Note

All claims expressed in this article are solely those of the authors and do not necessarily represent those of their affiliated organizations, or those of the publisher, the editors and the reviewers. Any product that may be evaluated in this article, or claim that may be made by its manufacturer, is not guaranteed or endorsed by the publisher.
